# Checklist and guidance on creating codelists for electronic health records research

**DOI:** 10.3310/nihropenres.13550.1

**Published:** 2024-04-17

**Authors:** Julian Matthewman, Kirsty Andresen, Anne Suffel, Liang-Yu Lin, Anna Schultze, John Tazare, Krishnan Bhaskaran, Elizabeth Williamson, Ruth Costello, Jennifer Quint, Helen Strongman

**Affiliations:** 1 London School of Hygine & Tropical Medicine, London, WC1E 7HT, UK; 2 Imperial College London, London, England, UK

**Keywords:** codelists, clinical codes, codesets, valuesets, electronic health records, checklist, reporting guidance, reproducibility

## Abstract

**Background:**

Codelists are required to extract meaningful information on characteristics and events from electronic health records (EHRs). EHR research relies on codelists to define study populations and variables, thus, trustworthy codelists are important. Here, we provide a checklist, in the style of commonly used reporting guidelines, to help researchers adhere to best practice in codelist development and sharing.

**Methods:**

Based on a literature search and a workshop with experienced EHR researchers we created a set of recommendations that are 1. broadly applicable to different datasets, research questions, and methods of codelist creation; 2. easy to follow, implement and document by an individual researcher, and 3. fit within a step-by-step process. We then formatted these recommendations into a checklist.

**Results:**

We have created a 9-step checklist, comprising 26 items, with accompanying guidance on each step. The checklist advises on which metadata to provide, how to define a clinical concept, how to identify and evaluate existing codelists, how to create new codelists, and how to review, finalise, and publish a created codelist.

**Conclusions:**

Use of the checklist can reassure researchers that best practice was followed during the development of their codelists, increasing trust in research that relies on these codelists and facilitating wider re-use and adaptation by other researchers.

## Plain english summary

When a person receives many types of health care, such as a doctor registering a diagnosis or prescribing a drug, information is collected in their computer system. This information is often organised in a structured way, so that each piece of information can be assigned a “code”. For example, if a person was diagnosed with type 1 diabetes, this could be recorded with the code E10 from the International classification of diseases, which contains codes on all possible diseases. For type 2 diabetes the code would be E11. To use this information for research, researchers need to define which people they want to study by making a list of all the relevant codes (a “codelist”). For example, to study people with type 1 and 2 diabetes they would need to include E10 and E11 in their codelist. The international classification of diseases coding system includes over 70,000 codes, and other medical dictionaries can include hundreds of thousands of codes. These lists can therefore be long and complex to create. While they are very important in ensuring that research using this data is correct, no step-by-step guidelines exist to help researchers create codelists. To tackle this, we created a checklist and guidance document which researchers can now use to make sure they don’t miss important steps and checks while creating their codelists, and to help them share their codelists so they can be re-used by other researchers. We collected recommendations that other authors have made before us, and developed detailed guidance together with experts in using these types of data for research.

## Background

Electronic health records (EHRs), containing data routinely collected for patient care, are commonly used for epidemiological research, bringing opportunities to address questions not easily answered with clinical trials or research-specific data collection ^1^. EHRs contain data structured and coded based on dictionary ontologies or clinical vocabularies. These vary widely in scope and specificity of coding; for example International Classification of Diseases ^2^ has traditionally been used for administrative purposes such as recording of deaths and hospital activity, whereas Systematized Nomenclature of Medicine – Clinical Terms (SNOMED CT) ^3^ was developed for use in clinical practice and includes a more extensive range of codes.

To extract meaningful information on health-related characteristics and events (e.g., diagnoses, prescriptions, referrals, test results, lifestyle factors, etc.) from EHRs, researchers create codelists (also referred to as clinical codelists, code sets, or value sets) ^4^. This is done by identifying relevant codes from the dictionary vocabulary (e.g. all the diagnosis, treatment, referral, etc. codes in SNOMED-CT indicating that a person has diabetes). In studies using EHRs, codelists define the study population, and other variables which researchers will use to answer the research question. Therefore, good practice in codelist development is an essential step in ensuring that codelists accurately capture the health-related characteristics or events of interest.

Checklists are increasingly being used in health research to promote adherence to recommended good practice ^5^, including EHR research where the REporting of studies Conducted using Observational Routinely-collected Data (RECORD) statement requires “a complete list of codes and algorithms used to classify exposures, outcomes, confounders, and effect modifiers” ^6^. While a number of articles already provide guidance on creating, sharing and managing codelists, these focus on specific scenarios (e.g. specific coding systems, or using specific codelist creation tools or methods), or pertain to higher level recommendations (e.g. for organisations, funders, or journals, rather than individual researchers) ^4, 7– 11^. Thus, we created an easy to use checklist and step-by-step guidance that can be used by EHR researchers to ensure good practice.

## Methods

### Checklist development

We formed a codelist task group including the following authors of this paper: JM, KA, AS, L-YL, and HS. All task group members were PhD students or academic staff members at LSHTM. The task group completed an initial literature search in PubMed to identify published papers describing methods and guidance for codelists. The most comprehensive review of the methodological literature on codelists was by Williams in 2017; this provides a set of best practice recommendations for future studies and software tools but did not aim to provide guidance for individual researchers on how to implement these recommendations ^4^. We updated this review, using the published search strategy, to find new literature released since 2017 (for a description of this literature search process see Box 1: Updated literature search). We also reviewed recommendations in other pertinent publications identified during this process ^8– 11^ and features of different codelist sharing websites and general purpose research repositories ^13– 16^.

**Box 1. Updated literature search**

We performed a literature search based on, and using the same search strategy as, the existing review by Williams R, *et al.*, 2017 ^4^ to find new literature released since 2017 on the topic. We did not intend to reevaluate recommendations proposed by Williams *et al.*, rather to identify important new literature on codelists that could be used to inform the creation of our checklist and guidance. We title-and-abstract-screened 427 papers published between June 2017 and December 2022 and indexed in PubMed, of which we full-text-screened 24. From these we excluded papers specifically discussing the transition in the US from ICD9 to ICD10, papers with a higher-level focus on terminologies such as mappings between them but no focus on codelists, and applied papers, including papers that use codelists but do not discuss construction, reuse, validation, or sharing of codelists (as was done in Williams R, *et al.*, 2017). There remained 9 papers from which we considered recommendations on codelist management. From these papers, we found 2 areas where additional recommendations we considered for inclusion in our checklist and guidance. The two identified topics are as follows:

1. When SNOMED CT is the available terminology, it may be preferrable to avoid “flat” codelists (i.e., a list of all codes to define a concept), in favour of using SNOMED CT concept hierarchies (i.e., a primary concept and its descendants optionally with additional relationships). These concept hierarchies may define more complex concepts (e.g. (Cerebrovascular accident OR History of Cerebrovascular accident) AND NOT Ruptured aneurysm) ^17– 19^. For drugs, it may be possible to use other terminologies such as MeSH, ATC, etc. to create similar concept hierarchies rather than creating “flat” codelists ^20^. While a recommendation to make use of concept hierarchies was already included in the Williams *et al.* 2017 review which was adapted for our checklist and guidance, we decided not to include guidance specific to the SNOMED-CT terminology, as this did not adhere to our criteria of being broadly applicable to different datasets, research questions, and methods of codelist creation.

2. If available, measures to check the quality of code sets should be made use of. The use of inter-terminology maps is recommended to check for codelists completeness when codelists exist in multiple terminologies (e.g. when creating a codelist in SNOMED CT, map an existing ICD-10 codelist to SNOMED and check for overlap and differences) ^21^. Some authors propose data centric natural-language processing methods to semi-automatically check codelists, however this will be dependent on the availability of such systems. ^22^ Within excluded papers, we found multiple recommendations for use of common data models which may address problems with codelists on a higher level, which we did not focus in this work. We mention the use of inter-terminology maps in the guidance section on searching for existing codelists.

Based on these publications and our expertise in using EHRs, the task group drafted an initial checklist, encompassing a set of recommendations on codelist development and sharing that needed to fit the following criteria: 1. broadly applicable to different datasets, research questions, and methods of codelist creation; 2. easy to follow, implement and document by an individual researcher; 3. fit within a step-by-step process where some items should be completed before others. This draft checklist was presented to, and pilot tested on example codelists in a workshop with a wider group of researchers in the Electronic Health records research group at the London School of Hygiene and Tropical Medicine (EHR research group). From this we gathered feedback which was used to further refine recommendations (for a description of this process, see Box 2: Feedback from workshop). Finally, we circulated the checklist to be reviewed and approved by the EHR research group at LSHTM and other stakeholders.

**Box 2. Feedback from workshop**

The task group convened a small group workshop to understand current codelist reporting practices and improve the process of creation, management, storage and sharing of codelists. All academic staff and PhD student members of the LSHTM Electronic Health Records research group were invited to attend. The workshop was held at the workplace for approximately 3 hours and was facilitated by the task group. Each of 4 groups with 3 to 4 people was provided with an example codelist (that had been employed in previous research), a draft version of the codelist guidance document based on a review of existing literature, and a questionnaire. Each group used the questionnaire to assess the codelist against the provided draft guidelines. Attendees were then asked to provide input to the draft guidelines in a plenary session. The plenary session was structured in two main discussion topics: existing codelists and new codelists. The discussion centred on key themes contained within these discussion topics. The task group took notes during discussions and collated notes from the filled-in questionnaires. Key themes for existing codelists included identifying published codelists and updating existing codelists. Key themes for creating new codelists included defining the clinical concept, creating the codelist, finalising the codelist and sharing the codelist. Several key takeaways emerged from these discussions:

1. Existing codelists: Participants stressed the need to create precise instructions for using previous codelists and updating them effectively. This would involve documenting instances of “absence of” evidence, for example, where no relevant codelists were found.
2. New codelists: Defining the clinical concept: Need for clear processes around defining the clinical concept. Participants advocated for clearly documenting and versioning iterative searches for synonyms and consulting experts early when defining the clinical concept. The participants stressed that these components should be part of the core documentation provided with the codelist and metadata.
3. Creating codelists: A suggestion was made to provide a cover sheet template to facilitate the implementation of information from the guidance.
4. Sharing codelists: Recognition of authorship: Participants emphasized the need to establish guidelines for recognizing and crediting individuals involved in codelist creation.
5. Improve knowledge about codelists and coding systems: The group advocated for an overview of codelists and coding systems to provide context and clarity in their usage.

In summary, the small group workshop discussions yielded valuable insights for enhancing codelist creation, and documentation practices, ultimately aiming to improve the clarity and effectiveness of these processes for better healthcare data management and research.

### Ethical consideration

Ethical approval was not required for this study as the current LSHTM policy is that only research activities involving human participants, their data, or their biological material must be submitted to and reviewed by the relevant LSHTM research ethics committee ^12^. The workshop is considered a professional involvement activity, and not participation in a study; therefore no informed consent is required. We also confirmed these with the LSHTM ethics team in their response "The current LSHTM policy is that only research activities involving human participants, their data, or their biological material must be submitted to and reviewed by the relevant LSHTM research ethics committee. Approval must be in place before the research starts. We do not expect to review literature reviews as there are no human participants, individual level human data, or biological material. We also do not expect to review public/professional 'involvement' activities. Involvement in research means research that is done 'with’ or 'by’ the people involved, not 'to', 'for' or 'about' them. It just allows people with relevant experience contribute to how research is designed, conducted and disseminated."

### Patient and public involvement

The target audience for this methods paper is researchers who use, or are planning to use, electronic health records for research. Researchers at all stages of their academic careers were involved throughout the project, including in developing objectives. We will involve researchers from a wider group of institutions by encouraging them to participate in the open review process. Patients or the public were not involved in this project.

## Results

Below we provide a 9-step checklist ( Table 1), comprising 26 items, with accompanying guidance on each step. We provide a filled-in example of the checklist in Table 2. 

**Table 1.** Checklist.

	Step No	Item	Information to be provided
Metadata			
Metadata	0	a. Name	*What is the name of the codelist?*
		b. Author(s)	*Who created the codelist?*
		c. Date finalised	*When was the codelist finalised?*
		d. Target data source	*What data is the codelist designed to be used with?*
		e. Terminology	*What is the terminology? (e.g., SNOMED, ICD)*
Define a clinical concept			
Define	1	a. Concept	*What is the clinical concept (e.g., the disease, drug, test result, etc…) of interest?*
		b. Timeframe	*Should the codelist capture new, current, and/or previous events?*
		c. Accuracy	*Should the codelist capture probable or definite codes?*
		d. Setting	*What is the (health care) setting (e.g., primary care, hospital care)?*
Identify and evaluate existing codelists			
Search	2	a. Sources searched	*Which sources were searched (e.g., internet search, codelist repositories)?*
		b. Existing codelists found	*Which suitable codelists did you find?*
Verify	3	a. Verified by others	*Which information is available to verify the quality of suitable codelists?*
		a. Verified by yourself	*Which checks did you conduct to verify the quality of suitable codelists?*
Reference	4	a. Existing codelists used	*Are you making use of any existing codelists? If yes, reference these, and* *specify how they are being used.*
Create a new codelist			
Prepare	5	a. Synonyms	*What are synonyms and related words for the clinical concept (e.g., different* *names for a disease/drug) and how did you identify these (e.g., source of* *clinical knowledge)?*
		b. Exceptions	*What should not be included in the codelist?*
Create	6	a. Method used	*Which method (e.g., a script, a tool) did you use to create the draft codelist?*
		b. Search terms	*Which search terms, and if applicable, exclusion terms did you use?*
		c. Hierarchy used to extend search	*Did you use a dictionary hierarchy (e.g., ICD-10 chapters, SNOMED-CT* *concepts) to modify your search? If yes, specify.*
		d. Decisions made while iterating	*Which decisions did you make while iteratively refining the draft codelist?*
		e. (Optional) Categories	*Did you specify subcategories within the codelist? If yes, specify.*
Review, finalise and publish			
Review	7	a. Reviewers	*Who reviewed the codelist and what expertise did reviewers have?*
		b. Scope of review	*What was reviewed (Just the draft codelist or also the method, terms, etc..)?*
		c. Evidence of review	*Where is the review process documented?*
Publish	8	a. Codelist published	*Where is the codelist published?*
		b. Resources published	*Where are the resources used to create the codelist (e.g., scripts, list of* *terms)?*

### Guidance

***Step 1: Define***

To find or create a suitable codelist, it is necessary to clearly state the following: Firstly, ( **1a - Concept**) state what the codelist intends to capture (e.g., a disease, drug, test results, etc..). Secondly, ( **1b - Timeframe**) state if current (prevalent), new (incident) or previous events are of interest (e.g., a codelist for incident asthma may only aim at capturing codes indicating a first occurrence of asthma not including asthma-related administrative or treatment codes which are likely to indicate ongoing asthma). Thirdly, ( **1c - Accuracy**) state if the codelist should prioritise sensitivity (i.e., includes codes “probably” indicating the clinical phenotype, e.g., “suspected asthma”, “referred to asthma clinic”) or specificity (e.g., includes codes that “definitely” match the concept)? Finally, ( **1d - Setting**) state where the codes occur (e.g. the health care setting such as primary care or hospital care and what types of codes are included e.g. diagnostic codes, referrals, administrative codes, disease history codes). Together, this information makes up a clinical concept (e.g., “codes definitely describing current or previous asthma in primary care, including diagnostic, treatment, administrative and disease history codes”).

***Step 2: Search***

**(2a – Sources searched**) Existing codelists that match your requirements can be identified (via an internet search (e.g., use a search-engine to search for “asthma codelist CPRD”), a search of publication databases, codelist repositories (e.g., the HDR UK phenotype library) or through existing collaboration and networks. Document which sources were searched. **(2b - Existing codelists found)** This search does not need to be systematic, but rather should identify codelists that may be directly reused or codelists that can help in creating a new codelist. To choose potentially suitable codelists, check the codelist metadata, including which clinical concept the codelist aims to capture, when the codelist was created, which database it was used in, which terminology, and which version of the terminology was used (as different versions of the same data source and terminology can contain different codes), and if there are any copyright restrictions. Codelists in other terminologies may also be useful, especially if these can be reliably mapped to the terminology of interest; however, this is not always possible. Document which suitable codelists you found.

***Step 3: Verify***

In addition to matching your requirements (in terms of concept, terminology, etc.) the quality of existing codelists needs to be verified. **(3a - Verified by others)** Identify which information is available, besides the metadata, to allow you to judge if the codelist was created using good practice. Projects or published studies dedicated to, or including codelist validation, may be of particular interest ^23^. ( **3b - Verified by yourself**) If available information isn’t sufficient to judge the quality of an existing codelist, various checks can be conducted depending on the specific use-case. The codelist may be cross-checked with other existing codelists to verify if different authors consistently include the same codes. A review of the existing codelist may be performed, similar as would be done for a newly created codelist (see Step 7). If you have access to your study data or the number of observations for each code, you may also check the number of records the codelist retrieves, which may be compared to expectations based on clinical knowledge or previous studies.

***Step 4: Reference***

( **4a - Existing codelists used**) Any existing codelists that are used should be referenced, giving credit to the author(s), and making it easy for others to evaluate your study, or find and adapt the codelist for their own purposes. You should reference whether you have identified a codelist that suits your purposes without modification, whether it required changes to be suitable for your study, or whether it was used to check or inform the creation of a new codelist, the existing codelist. You should also state what the existing codelist was originally used for. We suggest wording such as “codelist(s) for [clinical concept] are from/were adapted from/were cross checked with …”. References to existing codelist should include the author(s), year, and permanent identifier (such as a DOI, URL or manuscript reference). You may include these references directly as part of this checklist, in your study or codelist repository (see Step 8), or the section of your manuscript or manuscript appendix that describes study variables.

***Step 5: Prepare***

**(5a - Synonyms)** Identify synonyms and related words to the clinical concept (e.g., “asthma” for an asthma codelist; “stomach/gastric”, “cancer/neoplasm/malignant tumour”, etc., for a stomach cancer codelist; “beta-blocker”, “beta-adrenoceptor-antagonist”, and substance and trade names for a beta-blocker codelist). Consulting and referencing sources of clinical information can be useful. For example Medical Subject headings on PubMed ^24^, clinical knowledge summaries and guidelines (such as those provided by the National Institute for Health and Care Excellence (NICE) in the UK ^25^), and websites of patient organisations may all contain useful information. **(5b - Exceptions)** At this stage, identifying exceptions to the concept that shouldn’t be included in the codelist is also important (e.g., if only “allergic” forms of asthma should be included, identify the words “non-allergic”, “exercise-induced”, etc.). 

***Step 6: Create***

In this step, you create and iteratively refine a draft codelist. ( **6a - Method used**) This can be done in a variety of ways. Guidance on the use of specific methods for creating codelists is available elsewhere, including on using Stata scripts ^8^, online tools ^7^, and for specific use-cases, such as drug codelists ^10^. **(6b - Search terms)** Most approaches will involve searching a dictionary (also referred to as browser) firstly using search terms that correspond to the clinical concept or synonyms thereof, and secondly using exclusion terms to exclude codes that should not be in the codelist. For example, you create a script that searches for a list of predefined search terms (e.g., “asthma”, “inhaler”, etc..) and then exclude terms based on predefined exclusion terms (e.g., “referral”, “review”, etc..). Once finalised, report this list of search terms, and if applicable, exclusion terms. ( **6c - Hierarchy used to extend search**) Make use of dictionary hierarchies, e.g., through checking codes that are in the same or a descendant chapter as already included codes, to identify further codes that are related but may have different names or labels (e.g., check which other names for a disease or brand names for drugs may be included in the same Read code or ICD chapter or SNOMED-CT concept). **(6d - Decisions made while iterating)** When developing the draft codelist, the search should be iteratively refined by repeatedly checking the retrieved and excluded codes and adding terms to the list of search terms and exclusion terms. It may be better to also include codes where you are unsure if they should be in the codelist, as it is easier to exclude codes in the review stage than it is to add codes. Record important decisions made while refining the search, e.g., document the reasons for in- or exclusions. If necessary, revisit the definition of the clinical concept, and record additional decisions in descriptions or comments. ( **6e** - *Categories*) You may want to specify categories within the codelist, e.g., incident and prevalent codes, more sensitive or specific, only diagnosis codes or diagnosis and administrative codes, (e.g., allowing for the conduct of secondary or sensitivity analyses).

***Step 7: Review***

Your codelist, and how it was created, needs to be reviewed to check for omissions and mistakenly included codes. **(7a - Reviewers)** A suitable reviewer with relevant knowledge about your clinical concept of interest and experience of the health care setting of your study should be identified. Reviewers may be within your research group, or you may need to reach out to other researchers in the field (e.g., an asthma codelist may be reviewed by a general practitioner, asthma researcher or internal medicine physician). The actual review process can be handled in real time or asynchronously (e.g., via email or a GitHub issue thread). Having multiple reviewers that need to agree on the final codelist can further increase trust in the review process. **(7b - Scope of review)** The reviewer(s) should first read the description of the clinical concept, then, for each of the codes in the draft codelist, decide if the code is appropriate to include. Reviewing only the codelist, without reviewing the process of how it was generated risks missing codes that should be included; therefore, the method of how the codelist was created should also be reviewed. It is particularly important to give the full list of search terms and exclusion terms (e.g., are all terms included that could possibly refer to asthma?). Make sure to implement all the required changes and re-review if necessary. Whether or not to re-review is up to your judgment, but in general it will be more important when new search terms need to be added as compared to when only a few codes need to be dropped. **(7c - Evidence of review)** During the review process, interactions between the reviewer(s) and codelist creator(s) should be documented, e.g., via a GitHub Issue thread, or a spreadsheet where reviewers mark each code with yes/no or possible/probable/unlikely (e.g., “referral to asthma clinic”, may be marked as codes to be excluded, or codes to be included in a category of “possible asthma”).

***Step 8: Publish***

Finally, you should publish your codelist and metadata required by reporting guidelines such as RECORD. You should also publish resources used to create the codelist and related documentation to help readers to review, evaluate or reproduce your study, and reuse or adapt your codelist for future work. **(8a - Codelist published)** Codelists can be uploaded to general purpose repositories, ideally adhering to FAIR (Findable, Accessible, Interoperable, Reusable) principles ^26^. Examples of such repositories include zenodo.org or the Open Science Framework. You may also be able to adhere to FAIR principles when using your organisation’s research output repository, a GitHub or Gitlab repository, or uploading your codelist(s) as supplemental materials to your study. Codelists should be shared in a suitable format that is both human- and machine-readable (.txt, or .csv). **(8b - Resources published)** Share all resources used to create the codelist, such as search terms, scripts, and references, alongside the codelist. Depending on where the codelist is hosted, there may be predefined fields for metadata, or metadata can be included as part of the checklist.

## Discussion

We have developed a checklist to support the creation, adaptation, and re-use of high-quality codelists for research using EHR data, accompanied by step-by-step guidance. These were developed by researchers with relevant expertise and experience including members of the EHR research group at LSHTM, which has employed codelist based data extraction for hundreds of studies for a large range of health-related topics. In Table 2 we include an example of a filled in checklist.

**Table 2.** Example of filled in checklist.

	Step No	Item	Information to be provided
Metadata			
Metadata	0	a. Name	*Atopic eczema*
		b. Author(s)	*Julian Matthewman*
		c. Date finalised	*1 ^st^ January 2023*
		d. Target data source	*CPRD Aurum January 2023 release*
		e. Terminology	*SNOMED CT (mapped to CPRD MedCodeId)*
Define a clinical concept			
Define	1	a. Concept	*Atopic dermatitis/atopic eczema*
		b. Timeframe	*Current and previous*
		c. Accuracy	*Also including codes for unspecified forms of eczema that may be atopic*
		d. Setting	*Clinical records from UK primary care*
Identify and evaluate existing codelists			
Search	2	a. Sources searched	*Internet search, HDR UK phenotype library, LSHTM datacompass, opencodelists*
		b. Existing codelists found	*Identified a number of codelists but none for CPRD Aurum; one study describing validation of eczema codelists was found: Abuabara et al.* *2017 ( 10.1016/j.jid.2017.03.029)*
Verify	3	a. Verified by others	*See validation study above*
		a. Verified by yourself	*No further checks conducted as codelists could not be used directly*
Reference	4	a. Existing codelists used	*Medcodes from Abuabara et al. 2017 ( 10.1016/j.jid.2017.03.029) used to crosscheck new codelist*
Create a new codelist			
Prepare	5	a. Synonyms	*Identified from existing codelist, including Eczema, atopic dermatitis, Besnier's prurigo*
		b. Exceptions	Non-atopic forms of eczema as specified on the websites of the US ( https://nationaleczema.org/eczema/types-of-eczema/) and UK ( https://eczema.org/information-and-advice/types-of-eczema/) eczema societies
Create	6	a. Method used	*Used search terms and exclusion terms in a script while iteratively refining terms*
		b. Search terms	*Search terms: eczema, atopic dermatitis, besnier's prurigo, allergic dermatitis* *Exclusion terms: fh, family history, contact, dyshidrotic, neurodermatitis, nummular, seborrheic, stasis, asteatotic, discoid, ear, otitis,* *auditory canal, eyes, eyelid, facial, female genital, vulval, hand, male genital, pompholyx, dyshidrotic, scalp, seborrhoeic, cradle cap,* *varicose, gravitational, pustular, erythrodermic, infectious, psoriasis, psoriasiform, immunodeficiency, vesicular, friction, hyperkeratotic,* *venous eczema, lip licking, desiccation, papular, drug eruption, infective, craquele*
		c. Hierarchy used to extend search	*Checked for codes with the same SnomedCTConceptId and codes with a descendant Read code*
		d. Decisions made while iterating	*In addition to non-atopic eczema from the eczema society website, also identified other non-atopic forms and other irrelevant codes,* *including erythrodermic eczema (erythroderma), infectious eczematoid dermatitis (which is likely non-atopic), psoriasis, immunodeficiency* *syndromes, friction eczema, lip licking eczema, desiccation eczema, papular eczema, drug eruptions*
		e. (Optional) Categories	*Symptom and diagnosis codes only (i.e., no codes for referrals, drugs, history of, etc..), definite atopic eczema (i.e., no codes for eczema that* *is possibly atopic)*
Review, finalise and publish			
Review	7	a. Reviewers	*Julian Matthewman (clinician; conducted multiple studies on atopic eczema using UK primary care data), Sinéad Langan (dermatologist* *and expert on atopic eczema research using electronic health records)*
		b. Scope of review	*Both the draft codelist and search and exclusion terms were reviewed*
		c. Evidence of review	*The review process is documented in a GitHub issue thread at (…)*
Publish	8	a. Codelist published	*The codelist is published on LSHTM datacompass and the study GitHub repository*
		b. Resources published	*All resources are available at the study GitHub repository, including scripts and terms*

We expect these guidelines to be implemented by a wide range of institutions and research groups, including the EHR group at LSHTM. The guidelines can be used to train new EHR researchers, and develop or strengthen internal guidelines for publishing codelists. Developers of code list sharing platforms will also benefit from these guidelines to identify metadata that is required to allow codelists to be updated and reused. In comparison to previously published recommendations, the checklist and guidance here aim to be as universally applicable as possible, assuming as little as possible about the way of working, type of codelists to be created, type of terminology used, or tools used to create the codelist. As a consequence, it is not possible to cover every specific case in detail, therefore more narrow guidance may be useful. Examples of more specific guidance include guidance on creating drug codelists ^10^, SNOMED-CT codelists using concept hierarchies ^17– 19^, codelists using Stata scripts ^8^, codelists using the “termset” method ^7^.

The guidance was developed with more challenging coding systems in mind, such as SNOMED-CT and Read codes, which have a complex or overlapping hierarchical structures. The checklist is designed to cope with this complexity, however some steps of the codelist creation process in other settings (e.g. using only ICD coding) may be simplified.

This guidance underwent different validation steps ^27^, including a literature search, pilot testing and survey of peers. We have published the guidance in NIHR Open Research to support collaboration with the wider EHR community through open peer review, and to enable others to build upon the ideas presented here. Subsequent iterations, subject to funding, should involve pilot testing and input from larger groups of stakeholders, to ensure recommendations are useful for EHR researchers working in a range of different settings and on different topics.

## Conclusion

Codelists form the foundation of EHR research, however they may often be of suboptimal standard, not capturing what they are supposed to capture, and the way in which they are created and shared often precludes reuse and reproducibility. With this work, we provide a checklist, and step-by-step guidance, to help researchers adhere to best practice.

## Data Availability

Zenodo: Data for "Checklist and guidance on creating codelists for electronic health records research"; https://zenodo.org/doi/10.5281/zenodo.10852954 ^28^ This project contains the following data: Example codelist Questionnaire Data are available under the terms of the Creative Commons Attribution 4.0 International license (CC-BY 4.0).
