# Supplementary material for: Checklist and guidance on creating codelists for routinely collected health data research
Source: NIHR Open Res. 2024 Sep 18;4:20. Originally published 2024 Apr 17. [Version 2] doi: 10.3310/nihropenres.13550.2 (PMC11437289; doi:10.3310/nihropenres.13550.2)
Supplement: Peer review history

# Reviewer report: Reviewer response for version 2

**DOI:** 10.3310/nihropenres.14932.r32963

**Published:** 2024-10-04

**Authors:** Elizabeth Ford

**Affiliations:** 1 University of Sussex, Brighton, England, UK

I am happy to approve this version without reservations, the changes are sufficient for me.

Is the rationale for developing the new method (or application) clearly explained?

Yes

Is the description of the method technically sound?

Yes

Are the conclusions about the method and its performance adequately supported by the findings presented in the article?

Yes

If any results are presented, are all the source data underlying the results available to ensure full reproducibility?

No source data required

Are sufficient details provided to allow replication of the method development and its use by others?

Yes

Reviewer Expertise:

NA

I confirm that I have read this submission and believe that I have an appropriate level of expertise to confirm that it is of an acceptable scientific standard.

# Reviewer report: Reviewer response for version 2

**DOI:** 10.3310/nihropenres.14932.r32961

**Published:** 2024-09-27

**Authors:** Duncan Edwards

**Affiliations:** 1 University of Cambridge, Cambridge, England, UK

I am content with the amendments made in response to my review.

Is the rationale for developing the new method (or application) clearly explained?

Yes

Is the description of the method technically sound?

Yes

Are the conclusions about the method and its performance adequately supported by the findings presented in the article?

Yes

If any results are presented, are all the source data underlying the results available to ensure full reproducibility?

No source data required

Are sufficient details provided to allow replication of the method development and its use by others?

Partly

Reviewer Expertise:

General Practitioner using and generated electronic health records to manage the health of individuals and populations.  Clinical researcher using electronic health records.

I confirm that I have read this submission and believe that I have an appropriate level of expertise to confirm that it is of an acceptable scientific standard.

# Reviewer report: Reviewer response for version 2

**DOI:** 10.3310/nihropenres.14932.r32962

**Published:** 2024-09-27

**Authors:** Shirley Wang

**Affiliations:** 1 Howard Hughes Medical Institute - Harvard Medical School, Boston, Massachusetts, USA

The authors adequately addressed my comments

Is the rationale for developing the new method (or application) clearly explained?

Yes

Is the description of the method technically sound?

Yes

Are the conclusions about the method and its performance adequately supported by the findings presented in the article?

Yes

If any results are presented, are all the source data underlying the results available to ensure full reproducibility?

Partly

Are sufficient details provided to allow replication of the method development and its use by others?

Yes

Reviewer Expertise:

Phamacoepidemiology, meta-research

I confirm that I have read this submission and believe that I have an appropriate level of expertise to confirm that it is of an acceptable scientific standard.

# Reviewer report: Reviewer response for version 1

**DOI:** 10.3310/nihropenres.14709.r31892

**Published:** 2024-06-24

**Authors:** Duncan Edwards

**Affiliations:** 1 University of Cambridge, Cambridge, England, UK

- Is the rationale for developing the new method (or application) clearly explained?

Yes.  The need for improving the quality of codelists is well recognised by those involved in this area and this guidance is more robust and detailed guidance than I have seen before, which has tended to be in the form of expert opinion from smaller groups.  I suspect many researchers will immediately seek to embed this guidance in their groups' operating procedures.

- Is the description of the method technically sound?

Yes.  Iterative development and trialling from an expert group, was an appropriate method.  It's a weakness that the group contained only folk from LSHTM, though at least this fact is stated.

- Are sufficient details provided to allow replication of the method development and its use by others?

Mostly.  I am surprised that for 7b (or perhaps earlier) that some sort of incidence/prevalence check when a draft codelist is applied to a population is not explicitly included (i.e. comparing vs previous estimates in other studies, or versus clinical judgement by the reviewer of what a reasonable number and the gender/age breakdown would be) - in my experience this data exploration stage is where a lot of major bugs with codelists are found.  The description of 7b seems insufficiently detailed without explicit mention of this issue (though perhaps it is best covered elsewhere, I'm not sure 7b is the right place) - please consider a minor amendment.

- If any results are presented, are all the source data underlying the results available to ensure full reproducibility?

No relevant source data.

- Are the conclusions about the method and its performance adequately supported by the findings presented in the article?

Yes.  However, there is no mention of the subsequent use of codelists in clinical practice.  This is a huge issue in itself, and codelists developed based upon research papers can extremely swiftly be used in clinical practice to great population benefit (but also avoidable harm).  e.g. https://www.judiciary.uk/prevention-of-future-death-reports/alexander-reid-prevention-of-future-deaths-report/

 I'm not arguing that this paper should cover this issue (perhaps akin to the phase IV monitoring after drug trials) in great depth, because it is such a complex and important issue in its own right.  However, zero mention of the issue seems inappropriate and fails to highlight to researchers their role and responsibility in the subsequent use by others of their codelists.  So I would strongly suggest an amendment to the paper, albeit small, to mention this issue. Perhaps a paragraph in the discussion recognising the importance of phase IV monitoring of codelists subsequently used on the public, and the need for public, patient and frontline health worker engagement in developing best practice in this area too (perhaps the authors will tackle this in a subsequent paper!?).

Is the rationale for developing the new method (or application) clearly explained?

Yes

Is the description of the method technically sound?

Yes

Are the conclusions about the method and its performance adequately supported by the findings presented in the article?

Yes

If any results are presented, are all the source data underlying the results available to ensure full reproducibility?

No source data required

Are sufficient details provided to allow replication of the method development and its use by others?

Partly

Reviewer Expertise:

General Practitioner using and generated electronic health records to manage the health of individuals and populations.  Clinical researcher using electronic health records.

I confirm that I have read this submission and believe that I have an appropriate level of expertise to confirm that it is of an acceptable scientific standard, however I have significant reservations, as outlined above.

# Response

**Published:** 2024-09-19

**Authors:** Julian Matthewman

**Affiliations:** London School of Hygiene & Tropical Medicine, UK

- COMMENT 3.1: Is the rationale for developing the new method (or application) clearly explained?

Yes.  The need for improving the quality of codelists is well recognised by those involved in this area and this guidance is more robust and detailed guidance than I have seen before, which has tended to be in the form of expert opinion from smaller groups.  I suspect many researchers will immediately seek to embed this guidance in their groups' operating procedures.

*AUTHOR RESPONSE 3.1*: Many thanks for your helpful and positive comments. We will be very happy to see our guidance embedded in other groups’ operating procedures.

  

- COMMENT 3.2: Is the description of the method technically sound?

Yes.  Iterative development and trialing from an expert group, was an appropriate method.  It's a weakness that the group contained only folk from LSHTM, though at least this fact is stated.

*AUTHOR RESPONSE 3.2*: We agree that our manuscript and future work would be improved by wider collaboration. Future endeavors are, of course, subject to funding. We have made related changes to the manuscript under reviewer 1’s comments (4 and 5).

  

- COMMENT 3.3: Are sufficient details provided to allow replication of the method development and its use by others?

Mostly.  I am surprised that for 7b (or perhaps earlier) that some sort of incidence/prevalence check when a draft codelist is applied to a population is not explicitly included (i.e. comparing vs previous estimates in other studies, or versus clinical judgement by the reviewer of what a reasonable number and the gender/age breakdown would be) - in my experience this data exploration stage is where a lot of major bugs with codelists are found.  The description of 7b seems insufficiently detailed without explicit mention of this issue (though perhaps it is best covered elsewhere, I'm not sure 7b is the right place) - please consider a minor amendment.

*AUTHOR RESPONSE 3.3*: Your comment aligns with reviewer 1 comment 7; we have considered it in our response.

*AUTHOR CHANGE 3.3: see reviewer 1 comment 7.*

 If any results are presented, are all the source data underlying the results available to ensure full reproducibility?

 No relevant source data.

- COMMENT 3.4: Are the conclusions about the method and its performance adequately supported by the findings presented in the article?

Yes.  However, there is no mention of the subsequent use of codelists in clinical practice.  This is a huge issue in itself, and codelists developed based upon research papers can extremely swiftly be used in clinical practice to great population benefit (but also avoidable harm).  e.g. https://www.judiciary.uk/prevention-of-future-death-reports/alexander-reid-prevention-of-future-deaths-report/

 I'm not arguing that this paper should cover this issue (perhaps akin to the phase IV monitoring after drug trials) in great depth, because it is such a complex and important issue in its own right.  However, zero mention of the issue seems inappropriate and fails to highlight to researchers their role and responsibility in the subsequent use by others of their codelists.  So I would strongly suggest an amendment to the paper, albeit small, to mention this issue. Perhaps a paragraph in the discussion recognising the importance of phase IV monitoring of codelists subsequently used on the public, and the need for public, patient and frontline health worker engagement in developing best practice in this area too (perhaps the authors will tackle this in a subsequent paper!?). 

*AUTHOR RESPONSE 3.4*: Many thanks for alerting us to the potential use of research codelists in clinical care. We have now highlighted this issue in the discussion.

*AUTHOR CHANGE 3.4: added to discussion p16*: The guidance was developed with research as the use case; however codelists developed for research may end up being used in clinical practice. Further guidance, developed with public, patient, and healthcare worker input, is needed for a clinical care setting to maximize clinical benefit and prevent avoidable harm.

 Is the rationale for developing the new method (or application) clearly explained?

 Yes

 Is the description of the method technically sound?

 Yes

- COMMENT 3.5: Are sufficient details provided to allow replication of the method development and its use by others?

Partly

 AUTHOR RESPONSE 3.5: We believe that our methods are clearer following our review of the manuscript. For example, we added a summary of questionnaire responses to the manuscript.

 If any results are presented, are all the source data underlying the results available to ensure full reproducibility?

 No source data required

 Are the conclusions about the method and its performance adequately supported by the findings presented in the article?

 Yes

# Reviewer report: Reviewer response for version 1

**DOI:** 10.3310/nihropenres.14709.r31895

**Published:** 2024-06-12

**Authors:** Elizabeth Ford

**Affiliations:** 1 University of Sussex, Brighton, England, UK

This article describes the development of, and then presents, a researcher checklist to improve practice around code list development for EHR research. The aim is to improve practice of code list development, and reporting of code list meta data and origins. The article is very well written with good justification and explanation. The authors used reasonable checklist development methods (review, expert consensus, feedback workshop) and there are high levels of expertise in the team, with a strong track record within EHR research. Minor corrections to manuscript:

 Error in table 1: items under verify are labelled 3a and 3a – second item should be 3b. 

 In the example checklist, the name of the code list author is also given as the name of the reviewer. This seems to endorse the practice of self-review – however, the written guidance suggests getting outside help for review. Suggest change this to another name to encourage checklist users to seek an outside pair of eyes to review their checklist draft. 

 Top lines of page 9, second column “…whether it was used to check or inform the creation of a new codelist, the existing codelist.” Sentence does not make sense - please correct. 

 Suggestions for possible revision of the checklist: 

 All the items in the list are well thought out and well described in the guidance section. 

 Overall I endorse the checklist as it is, except could an item be included in the checklist on “has this codelist been trialled in the target data and numbers ascertained and sense checked against outside sources?”. 

 The reason I suggest this is that the NHS is putting a lot of funding into regional Secure Data Environments across England which will likely house novel datasets just being created from NHS data and providers for the first time – quality and coding culture and practices will largely be unknown. Taking and adapting off the shelf codelists from phenotype libraries seems like good practice, however, in our local experience, once patient lists ascertained via available code lists are compared against QOF returns, they are significantly under-ascertaining cases. Code lists must therefore be locally adapted. 

 So an additional checklist item which does not mandate but allows the option of reporting on what external sources the code list returns have been compared to, and how well it performed against these, would be useful for the next user of the code list and would encourage local teams to be cautious about applying code lists without checking them.

Is the rationale for developing the new method (or application) clearly explained?

Yes

Is the description of the method technically sound?

Yes

Are the conclusions about the method and its performance adequately supported by the findings presented in the article?

Yes

If any results are presented, are all the source data underlying the results available to ensure full reproducibility?

No source data required

Are sufficient details provided to allow replication of the method development and its use by others?

Yes

Reviewer Expertise:

Health data science, Electronic Health Records research, Data Governance, Public Engagement

I confirm that I have read this submission and believe that I have an appropriate level of expertise to confirm that it is of an acceptable scientific standard, however I have significant reservations, as outlined above.

# Response

**Published:** 2024-09-19

**Authors:** Julian Matthewman

**Affiliations:** London School of Hygiene & Tropical Medicine, UK

COMMENT 2.1: This article describes the development of, and then presents, a researcher checklist to improve practice around code list development for EHR research. The aim is to improve practice of code list development, and reporting of code list meta data and origins. The article is very well written with good justification and explanation. The authors used reasonable checklist development methods (review, expert consensus, feedback workshop) and there are high levels of expertise in the team, with a strong track record within EHR research. 

*AUTHOR RESPONSE 2.1*: Many thanks for reviewing and supporting our article, and for your considered advice.

**Minor corrections to manuscript:**

 COMMENT 2.2: Error in table 1: items under verify are labelled 3a and 3a – second item should be 3b. 

*AUTHOR CHANGE 2.2*: Done.

 COMMENT 2.3: In the example checklist, the name of the code list author is also given as the name of the reviewer. This seems to endorse the practice of self-review – however, the written guidance suggests getting outside help for review. Suggest change this to another name to encourage checklist users to seek an outside pair of eyes to review their checklist draft. 

*AUTHOR RESPONSE 2.3*: We have now changed the order of the two authors stated as reviewers so that the reviewer who is not the author of the codelist is stated first. In addition to getting outside help for the review, it may sometimes also be appropriate for the codelist author themselves to review the codelist if they have sufficient knowledge (which was the case in this example). Therefore we have kept the author of the codelist in the reviewer field, and additionally specified that they are the codelist author.

 AUTHOR CHANGE 2.3: *Table 2: Example of filled in Checklist, under Row 7 a (Reviewers)  changed to*: “Sinéad Langan (dermatologist and expert on atopic eczema research using electronic health records), Julian Matthewman (codelist author; clinician; conducted multiple studies on atopic eczema using UK primary care data)”

 COMMENT 2.4: Top lines of page 9, second column “…whether it was used to check or inform the creation of a new codelist, the existing codelist.” Sentence does not make sense - please correct. 

*AUTHOR CHANGE 2.4: *Deleted "the existing codelist" to correct the sentence.

**Suggestions for possible revision of the checklist:**

 COMMENT 2.5: All the items in the list are well thought out and well described in the guidance section. Overall I endorse the checklist as it is, except could an item be included in the checklist on “has this codelist been trialled in the target data and numbers ascertained and sense checked against outside sources?”. The reason I suggest this is that the NHS is putting a lot of funding into regional Secure Data Environments across England which will likely house novel datasets just being created from NHS data and providers for the first time – quality and coding culture and practices will largely be unknown. Taking and adapting off the shelf codelists from phenotype libraries seems like good practice, however, in our local experience, once patient lists ascertained via available code lists are compared against QOF returns, they are significantly under-ascertaining cases. Code lists must therefore be locally adapted. So an additional checklist item which does not mandate but allows the option of reporting on what external sources the code list returns have been compared to, and how well it performed against these, would be useful for the next user of the code list and would encourage local teams to be cautious about applying code lists without checking them.

*AUTHOR RESPONSE 2.5*: Thank you for this comment. We agree with you and encourage the reader to trial their codelist against internal and external data. Your comment aligns with reviewer 1 comment 1.7; we have considered it in our response.

*AUTHOR CHANGE 2.5: see reviewer 1 comment 7.*

# Reviewer report: Reviewer response for version 1

**DOI:** 10.3310/nihropenres.14709.r31896

**Published:** 2024-06-05

**Authors:** Shirley Wang

**Affiliations:** 1 Howard Hughes Medical Institute - Harvard Medical School, Boston, Massachusetts, USA

This paper describes a checklist for code list development and sharing of the code lists after creation. This is very important work, and the authors should be commended for the thoughtfully developed and comprehensive checklist for creating code lists. While the questionnaire and example code lists are provided in supplemental material, the results of the questionnaire that were used to support discussion and adaptation of the checklist are not.

 Minor comments:

 I would contend that this checklist applies not only to electronic health records research, but also to claims based research.

 All members of the task group and workshop were from LSHTM. However, the potential impact of this paper could have been far larger if the authors engaged members/perspectives outside of their own organization, from groups that have been thinking deeply about and already actively implementing processes for sharing code lists. For example, representation from groups like OHDSI, Sentinel, CPRD, N3C, etc. Such groups have experiences, preferences, and pipelines that may differ from experiences at LSHTM that would be helpful to inform what criteria would represent “good practice” for code list development. A task force gathering of experts could have been facilitated through a research society like the International Society for Pharmacoepidemiology or the International Society for Pharmacoeconomics Research. Just a thought for future work in this space.

 The recommendations are predicated on investigators intending to share code lists (which I very much support!) It would be helpful to have some discussion of the current prevalence of code list sharing (probably quite low). Do the authors have thoughts on how to encourage more routine citation of code lists? What other encouragement or culture shifts would be necessary for researchers outside LSHTM to routinely share their code lists?

 Code list creation based on mapping from other ontologies is mentioned. A little more discussion or cautionary words about the hazards of doing so given imperfect mappings and unmappable concepts could be helpful.

 Step 3 Verify. It would be helpful to emphasize more the importance of documenting performance characteristics of the code list (whether existing or new) or documenting the absence of such performance characteristics. If performance characteristics are available, then some details about the population in which it was validated would also be useful, for the user to understand how applicable those measurement characteristics would be to the population in which the code list is used.

Is the rationale for developing the new method (or application) clearly explained?

Yes

Is the description of the method technically sound?

Yes

Are the conclusions about the method and its performance adequately supported by the findings presented in the article?

Yes

If any results are presented, are all the source data underlying the results available to ensure full reproducibility?

Partly

Are sufficient details provided to allow replication of the method development and its use by others?

Yes

Reviewer Expertise:

Phamacoepidemiology, meta-research

I confirm that I have read this submission and believe that I have an appropriate level of expertise to confirm that it is of an acceptable scientific standard, however I have significant reservations, as outlined above.

# Response

**Published:** 2024-09-19

**Authors:** Julian Matthewman

**Affiliations:** London School of Hygiene & Tropical Medicine, UK

COMMENT 1.1: This paper describes a checklist for code list development and sharing of the code lists after creation. This is very important work, and the authors should be commended for the thoughtfully developed and comprehensive checklist for creating code lists.

*AUTHOR RESPONSE 1.1*: Many thanks for reviewing our manuscript and for your kind comments and expert advice.

 COMMENT 1.2: While the questionnaire and example code lists are provided in supplemental material, the results of the questionnaire that were used to support discussion and adaptation of the checklist are not.

*AUTHOR RESPONSE 1.2*: We have added the meeting minutes and transcribed questionnaire responses to the study’s online repository.

*AUTHOR CHANGE 1.2*: No changes to the manuscript necessary. The repository DOI cited in the manuscript represents all versions, and now resolves to the latest version of the repository.

**Minor comments**

 COMMENT 1.3: I would contend that this checklist applies not only to electronic health records research, but also to claims based research.

*AUTHOR RESPONSE 1.3*: We agree that the checklist applies to all routinely collected database studies that require the generation of codelists to answer the study question, especially those that use complex dictionaries or ontologies such as SNOMED or ATC codes that are used in many databases worldwide. We changed our terminology throughout the manuscript, and we are now using the term “routinely collected health data” instead of “electronic health records” to emphasise that this checklist applies to all types of routinely collected health data including claims data as well.

*AUTHOR CHANGE 1.3: *We changed the term “electronic health records” into “routinely collected health data” throughout the manuscript.

 COMMENT 1.4: All members of the task group and workshop were from LSHTM. However, the potential impact of this paper could have been far larger if the authors engaged members/perspectives outside of their own organization, from groups that have been thinking deeply about and already actively implementing processes for sharing code lists. For example, representation from groups like OHDSI, Sentinel, CPRD, N3C, etc. Such groups have experiences, preferences, and pipelines that may differ from experiences at LSHTM that would be helpful to inform what criteria would represent “good practice” for code list development. A task force gathering of experts could have been facilitated through a research society like the International Society for Pharmacoepidemiology or the International Society for Pharmacoeconomics Research. Just a thought for future work in this space.

*AUTHOR RESPONSE 1.4*: Our working group initially aimed to recommend an existing codelist bank for everyone to use or develop a codelist bank for LSHTM’s EHR group. We quickly realised how complex and ambitious this task was, especially given the lack of allocated funding or time.  We therefore prioritised developing broad internal recommendations for codelist creation and sharing that are applicable to multiple use cases. We have shared this work in our manuscript.

 A future mission is to improve the quality and availability of codelist banks. We are open to working with a wider group of experts to secure funding for this work, and agree that this would increase the relevance and impact of our work

*AUTHOR CHANGE 1.4: see comment 5 below as the changes are linked.*

 COMMENT 1.5: The recommendations are predicated on investigators intending to share code lists (which I very much support!) It would be helpful to have some discussion of the current prevalence of code list sharing (probably quite low). Do the authors have thoughts on how to encourage more routine citation of code lists? What other encouragement or culture shifts would be necessary for researchers outside LSHTM to routinely share their code lists?

*AUTHOR RESPONSE 1.5 *: Thank you for your comment. There has been a welcome recent push towards open science and reproducibility by publishing both programming code and codelists. A requirement for authors to publish all programming code and study code lists with any manuscript might help to create a culture of routinely sharing code lists. Additionally, encouraging data providers and research organisations to establish and maintain accessible repositories for these resources would further enhance this. An example of such an initiative is OpenSAFELY and OpenCodelists, where the sharing of codelists and programming code is an integral part of the study workflow. These platforms facilitate reproducibility and improve the overall transparency of medical research.

*AUTHOR CHANGE 1.5: Added discussion (p16)*: While codelists are shared alongside the majority of studies that use them (a recent review found about 70% reported at least one diagnostic or treatment code), the resources used to create these codelists are rarely shared ^28^. Besides journals necessitating (as with analytical code) ^29,30^ that codelists be published alongside manuscripts, data providers and research organisations should be encouraged to establish and maintain repositories that facilitate sharing of more complete codelist information. Future research may review current codelist banks with a view to improving the completeness of information captured.

 COMMENT 1.6: Code list creation based on mapping from other ontologies is mentioned. A little more discussion or cautionary words about the hazards of doing so given imperfect mappings and unmappable concepts could be helpful.

*AUTHOR RESPONSE 1.6*: We have added some words of caution describing the challenges of mapping between dictionaries and ontologies.

*AUTHOR CHANGE 1.6: (underlined text added to* *Methods p6)*:

 The use of inter-terminology maps is recommended to check for codelists completeness when codelists exist in multiple terminologies (e.g. when creating a codelist in SNOMED CT, map an existing ICD-10 codelist to SNOMED and check for overlap and differences).(20) However, caution is needed when mapping terms from different ontologies as they may have been created for different purposes (e.g., documentation, billing, registries, referrals or information sharing) and are often used in different care settings (e.g., SNOMED CT in primary care in the UK and ICD-10 codes in secondary care).

 COMMENT 1.7: Step 3 Verify. It would be helpful to emphasize more the importance of documenting performance characteristics of the code list (whether existing or new) or documenting the absence of such performance characteristics. If performance characteristics are available, then some details about the population in which it was validated would also be useful, for the user to understand how applicable those measurement characteristics would be to the population in which the code list is used.

*AUTHOR RESPONSE 1.7*: Thank you for this comment which links to comments from other reviewers. In alignment with Herrett el al.’s (2010) publication on validation methods of diagnoses in CPRD, we added an additional step to the checklists (Step 8 – Checks) which offers the opportunity to comment any internal or external validation steps. Further, we expanded in the main text on what these internal and external validation steps might look like.

*AUTHOR CHANGE 1.7A (Added step 8 to Table 1p9)*:

 Check (8)

 a. Internal checks

*What method(s) were used for internal checks, if any, and what are the findings?*

 b. External checks

*What method(s) were used for external checks, if any, and what are the findings?*

*AUTHOR CHANGE 1.7B (Explanation added to Results p14):*

 Where possible, code lists should be checked against the database they were created for (internal) or other data sources (external). Internal methods within the intended database could include the reporting of the numbers of individuals who were identified with the clinical concept of interest and potential sensitivity analyses comparing different versions of the code list with different inclusion/exclusion criteria applied. External checks could consist of a comparison of prevalence and incidence measured within the dataset to that published in external literature, or a validation study involving primary data collection to estimate the sensitivity, specificity or positive predictive value of a certain code list. More detail on potential options for conducting a code list validation can be found in a previous publication (Herrett et al.).

*AUTHOR CHANGE 1.7C: (Added step 8 to Table 2, example of filled in checklist, p18)*

 Check (8)

 a. Internal checks

*Checked the number of observations in the CPRD Aurum (2023/03) code browser for the different categories:*

*full codelist: 17.4 million*

*diagnosis and symptom codes: 16.8 million*

*definite atopic eczema: 6.4 million*

 b. External checks

*No checks were performed using external data*
